# Supplementary material for: Psychological experience of patients with postpartum depression: A qualitative meta-synthesis
Source: PLoS One. 2024 Nov 6;19(11):e0312996. doi: 10.1371/journal.pone.0312996 (PMC11540214; doi:10.1371/journal.pone.0312996)
Supplement: S1 File — (DOCX) [file pone.0312996.s001.docx]

**S1 File Search formula using PubMed as an example**

#1："Pregnant Women"[MeSH Terms]

#2："woman pregnant"[Title/Abstract] OR "mothers"[Title/Abstract] OR "parturients"

[Title/Abstract]

#3：#1 OR #2

#4:"depression, postpartum"[MeSH Terms]

#5："postnatal depression"[Title/Abstract] OR "depression postnatal"[Title/Abstract] OR

"post partum depression"[Title/Abstract] OR "postpartum depression"[Title/Abstract] OR "post natal depression"[Title/Abstract]

#6：#4 OR #5

#7："Emotions"[MeSH Terms]

#8："experience"[Title/Abstract] OR "feeling"[Title/Abstract] OR "need"[Title/Abstract]

OR "demand"[Title/Abstract] OR "attitude"[Title/Abstract]

#9：#7 OR #8

#10:"Qualitative Research"[MeSH Terms]

#11:"descriptive analy*"[Title/Abstract] OR "interview"[Title/Abstract] OR "content

analy*"[Title/Abstract] OR "thematic analy*"[Title/Abstract] OR "grounded theory"

[Title/Abstract] OR "phenomenology"[Title/Abstract] OR "qualitative study"

[Title/Abstract]

#12:#10 OR #11

#13:#3 AND #6 AND #9 AND #12
