# Supplementary material for: Psychological experience of patients with postpartum depression: A qualitative meta-synthesis
Source: PLoS One. 2024 Nov 6;19(11):e0312996. doi: 10.1371/journal.pone.0312996 (PMC11540214; doi:10.1371/journal.pone.0312996)
Supplement: S2 File — (DOCX) [file pone.0312996.s002.docx]

We used meta-aggregation to synthesize the results of the qualitative study. The results of the literature were integrated using the pooled integration method recommended by the JBI Center for Evidence-Based Health Care. Guided by qualitative research methods, researchers repeatedly read, deeply dissected, and interpreted the findings of the included literature and formed new sub-themes after combining similar findings. The sub-themes with certain connections were then synthesized into a new integrative theme, and the corresponding sub-themes were assigned to the integrative theme. The two researchers repeatedly read, analyzed, and compared 28 pieces of literature to distill a total of 51 findings. They grouped similar findings into 12 sub-themes and synthesized them into three descriptive themes, with no mutual exclusivity in the categorization of findings(S2 File)

**S2 File** **Data Extraction and Integration Process**

| **Descriptive themes** | **Sub-themes** | **Extracted results** | **Source** |
| --- | --- | --- | --- |
| Negative physical and psychological experiences and coping  strategies | Negative Psychological Experiences | Sad, repressed psyche | *"I don't know why, I just want to cry."**[Tian et al, 2018]* |
|  |  | Poor concentration, lack of joy and confidence in life | *"I used to love ... but now they don't make me happy, and I'm losing interest in these hobbies."* *[Zhao, 2018]* |
|  |  | Frustration | *"Yeah, that was another thing I was blaming myself for. I can**'t even feed my own baby." [Tyson et al, 2020]*  *I'm a strong woman. I used to be able to do everything well, but now when the baby cries, I'm really at a loss![Tian et al, 2018]* |
|  |  | Lonely | *"I had gotten fat and was unhappy about it. My husband did not show any interest in me. I felt that my husband was growing more distant from me."[Kazemi et al, 2018]* |
|  |  | Helpless | *"I can't control it; it's a terrible thing. I feel trapped. I feel like there's absolutely no way to get rid of this clock. No matter how hard I try, these terrible feelings won't disappear."**[Beck et al, 1992]* |
|  |  | Significant changes in mood | *"I can't control it; it's a terrible thing. I feel trapped. I feel like there's absolutely no way to get rid of this clock. No matter how hard I try, these terrible feelings won't disappear.**"[Beck et al, 1992]* |
|  |  | Psychology of conflict | *"Mothers also bore the burden of guilt over not giving their babies the love they felt the babies needed.The women feared that psychologically they were harming their infants."* *[Beck et al, 1992]* |
|  |  | Fear of not being able to resume a normal life | *"My big fear.. I wasn't going to be the same person I was before the experience; that I would never quite get over it."[Beck et al, 1992]* |
|  |  | Close oneself off | *"Looking at a flabby waist and a bloated face, I prefer to isolate myself from others."* *[Zhao, 2018]* |
|  |  | Frustrated and pessimistic | *"But there were times when it was like the baby was crying. So what? I can't deal with this. Can't deal with it..." [Amankwaa et al, 2003]* |
|  | Physical burden | Physical pain (e.g. wound pain) | *"I had a normal labor, but the baby's head was too big to be born, so I had a C-section. Now I can't turn over because of the pain in the incision." [Liu et al， 2022]*  *"After delivering, my breasts started swelling. I tried using herbal medicine, but all in vain. I decided to go to… Right now, I use only one breast to feed my child and also buy milk because milk from one breast is always not enough..."* *[Atuhaire et al, 2021]* |
|  |  | Breastfeeding difficulties | *"After delivering, my breasts started swelling. I tried using herbal medicine, but all in vain. I decided to go to… Right now, I use only one breast to feed my child and also buy milk because milk from one breast is always not enough..." [Atuhaire et al, 2021]* |
|  |  | mother's milk is scarce | *"My doctor said that I had a decrease in lactogen production from my pituitary gland due to neuroendocrine changes, and although I didn't really understand the terminology, I just rejected breastfeeding in particular, and could barely get emotional attachment from my child."[Zhao, 2018]* |
|  |  | Difficulty falling asleep or insomnia | *"I'm so tired, I'm so tired every day; hey, I'm in a hurry at home; when I first gave birth, I was so annoyed with her (the baby), and the baby cried and fussed, I was so annoyed. At that time, I was sleepy, tired and had no sleep."[Zhang et al, 2019]* |
|  |  | Spirit weary, strength exhausted (idiom); spent | *"I felt tired all the time, I still do. Exhausted, actually. I can't pull myself out of it. I am so tired most of the time and have great difficulty staying awake. I even bought a book, what's it called, on babies, but I don't seem to be able to read even a paragraph on some days." [Barr, 2008]* |
|  | Self-recovery from postpartum depression | Seeking help | *"I never got any help, really. I would just call people that I knew. I had received counseling, marriage counseling prior to the birth of my second baby... And I would call my—my—she was a psychiatrist. I would call her occasionally during the course of that first year." [**Amankwaa et al, 2003]*  *"... Just like now, this group discussion, we are all strangers, but it feels very therapeutic." [Hanach et al, 2024]* |
|  |  | Enhancement of cognition | *"…You know; we the Muslims listen to Quran. During this terrible time, I engaged myself more in prayers and reading of the Quran as this makes me feel better…. Usually, when I was in a very bad mood, I would first cry, but quickly resort to praying or reading of the Quran which would relieve and I feel better."[**Atuhaire et al, 2021]* |
|  |  | Self-motivation | *"But then you have to recover; you have no choice after a while. I think once I started feeling like my old self, you have to say, "Ah, I don't want to feel like this anymore." And really just get your head back in the game. Um, I think it is up here (pointing to forehead) as far as just trying to change yourself. The way you look at it, not be ashamed of it, not, you know." [Williams, 2013]* |
|  |  | Emotional regulation | *“I was so lost I didn’t care who was treating me, I just wanted somebody that understood. ... Later in the day I’d try, I could change the baby, I could do that. I could change her, you know and they were right here, in case I freaked.”**[Williams, 2013]*  *"I controlled my nurturing. Um, it would be more just trying to make me happy. Um, just doing things that I liked."**[Williams, 2013]* |
|  |  | Divert attention | *"... once I got out and made myself do it, it would kinda take my mind off of it.”**[Williams, 2013]* |
|  |  | Spiritual needs | *“Ooooh, just the medicine, went and saw the doctor, spent time with my family a lot, prayed. Went to church, got myself out of the house. ...went to a lot of mothering groups, stuff like that, Bible study.”[Williams, 2013]* |
|  |  | Adherence to postnatal rehabilitation exercises | *“Yes, so something in the house that is ready to use. Absolutely I would use it because it’s there and you know you’re not going anywhere. You’re not leaving the house and basically, it’s the only way I can see that it’s quite an easy way of exercising for new mums.” [Maria et al, 2021]* |
|  |  | Reduced screen time | *"I think that social connections are really important because that's when screen time becomes a factor, especially when you're feeling isolated or alone. So, if you're able to encourage more group activities or social interactions, such as partnering up with someone, that would be a really good way to reduce screen time."* *[Maria et al, 2021]* |
|  |  | Ending an unfortunate marriage | *"At first, I had thoughts of killing myself. Th**Williams[31],*  *2013en, I decided that instead of killing myself, I would rather leave this marriage. I would rather look for work as a housegirl, earn some money that would take care of us, rather than keep suffering in his house to the extent of wanting to kill myself." [Atuhaire et al, 2021]* |
| Role transition discomfort and impact | Role transition | Discomfort in role | *"Just gave birth to a child, I was very anxious. I have very serious hemorrhoids and pubic symphysis separation. The first night back from the hospital, the lower half of my body felt crippled, and I couldn't move. I experienced severe pain and had to call '120' to go to the hospital ... I just gave birth to a child, and I'm not adapting well to the feeling of nightmares." [Zhang et al, 2019]* |
|  |  | Conflict of roles | *"I feel like I'm still a kid myself and can't take care of my kids' food, drink, and sleep."* *[Tian et al, 2018]* |
|  |  | Role Adaptation | *"Well, we wanted her very much, and I knew it would be hard having a baby. I knew that it would take a little while to get into a routine and things would change, but I didn't think things would be as disrupted as they have been. I expected to be able to do most things I used to do pretty much straight away." [Buultjens et al, 2007]* |
|  | Life-style modification | The old way of life has been disrupted | *"Since having a baby, I feel like my life can be described as a military mess (bitter smile)."* *[Tian et al, 2018]* |
|  |  | liberty is restricted | *"It's hard to think about the days ahead. It's hard to go out to dinner with friends when you have kids, not to mention going out to play."[Tian et al, 2018]* |
|  |  | Influencing the personal development process | *"Since then, it has been difficult to find another job. I solely depend on my husband, who has failed to provide us with basic needs like milk, clothes, food, and even paying the house rent… All this just stresses me out." [Atuhaire et al, 2021]* |
|  |  | Life is dull and tasteless | *"In the past, I always like to get together with my friends to chat and hang out, but now I prefer to be alone and don't want to hang out with other people, and over time, I even have some horrible thoughts, what's the point of living like this all day long, why don't I just die?"* *[Zhao, 2018]* |
|  |  | Decline in living standards | *"I insisted on a normal labor just to spend less money, and frankly, after all, unlike others who have health insurance, I still ended up in an incubator, which costs too much per day."[Liu et al, 2022]* |
|  | Family life events | Husband-wife relationship | *"My family used to revolve around me, but now the whole family revolves around the children, and everything is centered on the children (despondent look)."[Tian et al, 2018]*  *"When my husband gets angry, he uses physical violence against me and always verbally insults me."【Kazemi et al, 2018】* |
|  |  | Ineffective communication | *‘‘When he is in bad mood, you can get the idea that he doesn’t like me, that he doesn’t care about me … that I am merely a milk-cow…’’[Edhborg et al, 2005]*  *"He would have said earlier, my in-laws have nothing to do, just stay at home, and then I cook for him every day, and did not even come."[Liu et al, 2019]* |
|  |  | Relations between mother-in-law and daughter-in-law | *"As soon as I heard that I had depression, my in-laws stayed away from me, and they didn't even care about my children, thinking that they would inherit my disease as well. I don't think they see me as a daughter-in-law at all."[Zhao, 2018]* |
|  |  | Living customs | *"My mother-in-law is from Shandong, and I'm from Hunan. The diet is different, but she only cares about her son eating well. I sit on the moon every day, eating steamed buns and noodles. The dishes are cooked very salty, and I can't eat them!" [Wang et al, 2019]* |
|  |  | Parenting Thinking | *"My mother-in-law is always telling us that you're spoiling the baby, you always have to hold him."[Zhao, 2018]* |
|  | Impact of parenting | Ignoring your health needs | *"I just felt that I needed to suppress my needs to ensure that [my daughter] was taken care of..." [Borrero et al, 2024]*  *"I'm very against medicine... um, I knew that I needed to do what was best for my older daughter. And I felt that my mood was affecting her mood." [Borrero et al, 2024]* |
|  |  | Had to make some tough decisions. | *"...We did it with our first, so that actually hasn't been hard. Um, it has been easier the second time around. The decision to take, um, I am on an antidepressant. The decision to take that was hard."* *[Borrero et al, 2024]* |
|  | Socio-cultural influences | Traditional custom | *"...She questioned why I couldn't take care of the baby myself. She said that the baby was my own son, and I am the mother. Why didn't I care for him myself? She meant I didn't take up my own responsibility..." [Leung et al, 2005]* |
|  |  | Gender bias | *"For the baby's gender, they look a little heavy, held in the heart. I am more or less in a bit of pain. I did not want a second child at that time. Their families very much wanted a boy, and now they are also very unhappy because of the birth of a daughter. If it were a boy, it would have been better, and the relationship between the mother-in-law and daughter-in-law would have been better!" [Zhang et al, 2019]* |
|  |  | "Doing the month" | *"The 'doing the month'... It is like being in prison for me to be confined at home. I was not allowed to do anything but lie in bed. It was so boring. You know, I was a career woman before the baby was born, but..." [Gao et al, 2010]* |
| Lack of relevant support | Lack of relevant support | Family's lack of understanding and concern | *"Otherwise, I do most of the work like washing, cooking, sweeping, and mopping myself. And when I feel tired and sometimes fail to eat…Yes, that's when I think that maybe I should leave or sleep the whole day, but the baby's clothes need to be washed. Yet, my husband can never support me…not even to hold the baby like I do." [Atuhaire et al, 2021]*  *"I think in the postpartum period, my husband and I were particularly low on the emotional side, and he just put the kids first. I was separated from the pubic symphysis at that time, so I felt like I was paralyzed, and I couldn't move from the waist down with severe pain, and the orthopedic surgeon at the hospital said that he wanted to take a film, and when my husband heard that it would have an effect on breastfeeding, he said, 'That won't work,' and he didn't allow the film to be taken. At that time, I was furious ...... I had just had a baby and was not used to it, it felt like a nightmare, so I especially wanted to go to work!"[**Zhang et al, 2019]* |
|  |  | Lack of understanding and prejudice on the part of professionals | *"I got answers from professionals like, 'There is nothing wrong with you, go back home and stop disturbing us. Basically, you are wasting our time.' And they were horrible. It was a doctor who said that to me. My husband was sitting with me that day as well. I don't know if they would have said that if I were white." [Wittkowski et al, 2011]* |
|  |  | Lack of peer support | *"When my mood is depressing, I sometimes think that if I can meet a few moms who are in the same trouble as me and talk to each other, I might feel better. If you can organize some of these events, I'd love to come!" [Wang et al, 2019]*  *“One thing that I felt like I needed and I didn’t have was a network of other moms. I am in a country where I am an expat and I work from home. I haven’t been here for very long. I don’t have any friends here, really. So I don’t have a network of other moms that I speak to or can troubleshoot or can be like a listening point.”**[Hanach et al, 2024]* |
|  | Lack of practical support | Specific real-life challenges and needs | *"...The first 40 days after you give birth, you go back to your mom's house. So, when I gave birth, I was at my mom's for 40 days. I had a lot of help from my mom and my sisters. They would cook the food." [Hanach et al, 2024]*  *“It’s part of our culture, the first 40 days after you give birth, you go back to your mom’s house. So when after I gave birth, I was at my mom’s for 40 days. I had a lot of help from my mom and my sisters. They would cook the food."[Hanach et al, 2024]*  *"...The first 40 days after you give birth, you go back to your mom's house. So, when I gave birth, I was at my mom's for 40 days. I had a lot of help from my mom and my sisters. They would cook the food." [Tian et al, 2018]*  *"My husband is an engineer and is very research-based, so he really likes articles and whatnot and likes to know the details of how the vaccination is created. He's really interested in that. I've really relied on him for that research piece of it."* *[Borrero et al, 2024]*  *"My husband’s aunt is also a lactation consultant, so I, um, text her a lot with questions. So handy…And then, my sister had a baby in June, so we are constantly just talking about baby things, and my mom had five children. And so, I’m constantly asking her, um, questions about the baby."**[Borrero et al, 2024]*  *"I think part of the problem with lack of providers being able to spend time with moms postpartum is that the easy fix is saying, let’s, let’s put you on some meds rather than necessarily like doing therapy or like figuring out what the problem is and offering some solutions."[Borrero et al, 2024]* |
|  | Lack of information support | Lack of knowledge about postpartum depression | *"I don't know anyone who has it (PND in Nigeria) because I don't have it. I didn't have it in Nigeria. I never knew it existed...do you understand? Until I came to this place." [Gardne et al, 2014]* |
|  |  | Lack of knowledge about infant care | *"I wonder if the baby's jaundice is better. Is he still on the blue light? How long will the treatment take?" [Li et al, 2015]* |
|  |  | Lack of knowledge about self-care | *"I had a cesarean section. I don't know if it's because of the anesthesia on my waist. After I came home from the hospital, I suddenly had to lie in bed for several days and couldn't move. My husband had to help me turn over, which was too painful. My mood was particularly low during those days. If I had known that I might be in such a situation, I would have paid attention to it myself!" [Wang et al, 2019]*  *"I've heard that doing postnatal exercises can shape your body without harming it, but I don't know how to do it, so I really hope someone will guide me!" [Tian et al, 2018]* |
|  | Lack of other support | Lack of practical action support, e.g. financial, material, career | *"suffer hardships" and "live hand to mouth." [Edhborg et al, 2015]*  *"I was in charge of the company's human resources before, but now I can't go back to work for more than half a year, so I'm sure this piece will be handed over to him."[Tian et al, 2018]* |
|  |  | Lack of support from specialized institutions, e.g., psychological counselling services | *"There is a huge stigma of being mentally ill in the public, but for us Asians, there is a double disadvantage. I really fear that work will find out." [Wittkowski et al, 2011]* |
